# Enhancement of Wound Healing by Non-Thermal N_2_/Ar Micro-Plasma Exposure in Mice with Fractional-CO_2_-Laser-Induced Wounds

**DOI:** 10.1371/journal.pone.0156699

**Published:** 2016-06-01

**Authors:** Pei-Lin Shao, Jiunn-Der Liao, Tak-Wah Wong, Yi-Cheng Wang, Steve Leu, Hon-Kan Yip

**Affiliations:** 1 Department of Materials Science and Engineering, National Cheng Kung University, Tainan 70101, Taiwan; 2 Medical Device Innovation Center, National Cheng Kung University, Tainan 70101, Taiwan; 3 Department of Dermatology, Department of Biochemistry and Molecular Biology, Medical College and Hospital, National Cheng Kung University, Tainan 70101, Taiwan; 4 Center for Translational Research in Biomedical Sciences, Kaohsiung Chang Gung Memorial Hospital and Chang Gung University College of Medicine, Kaohsiung 83301, Taiwan; 5 Division of Cardiology, Department of Internal Medicine, Kaohsiung Chang Gung Memorial Hospital and Chang Gung University College of Medicine, Kaohsiung 83301, Taiwan; University Paul Sabatier, FRANCE

## Abstract

Micro-plasma is a possible alternative treatment for wound management. The effect of micro-plasma on wound healing depends on its composition and temperature. The authors previously developed a capillary-tube-based micro-plasma system that can generate micro-plasma with a high nitric oxide-containing species composition and mild working temperature. Here, the efficacy of micro-plasma treatment on wound healing in a laser-induced skin wound mouse model was investigated. A partial thickness wound was created in the back skin of each mouse and then treated with micro-plasma. Non-invasive methods, namely wound closure kinetics, optical coherence tomography (OCT), and laser Doppler scanning, were used to measure the healing efficiency in the wound area. Neo-tissue growth and the expressions of matrix metallopeptidase-3 (MMP-3) and laminin in the wound area were assessed using histological and immunohistochemistry (IHC) analysis. The results show that micro-plasma treatment promoted wound healing. Micro-plasma treatment significantly reduced the wound bed region. The OCT images and histological analysis indicates more pronounced tissue regrowth in the wound bed region after micro-plasma treatment. The laser Doppler images shows that micro-plasma treatment promoted blood flow in the wound bed region. The IHC results show that the level of laminin increased in the wound bed region after micro-plasma treatment, whereas the level of MMP-3 decreased. Based on these results, micro-plasma has potential to be used to promote the healing of skin wounds clinically.

## Introduction

The disruption of the normal anatomic structure and function of skin or organ tissues results in the formation of a wound [1]. Wounds affect over 6 million people in the US at an annual cost of $25 billion [2,3]. Acute skin wounds undergo a repair process that leads to benign scars. Failure of this process, due to the wound area and/or depth exceeding the patient’s ability to heal, may lead to an undesirable scar or a chronic or non-healing wound [1,4]. Chronic and non-healing wounds are especially costly because they require repetitive treatments; for example, a diabetic foot ulcer typically costs $50,000 to treat [5]. Chronic wounds affect 1% of the population at any given time [6]. Thus, an approach that improves wound healing could have considerable economic and personal benefits. Although skin allografts, xenografts, or tissue-engineered skin substitutes have been proposed for wound treatment, drawbacks include limited availability of donor tissue, rejection by the host’s immune system, and high cost [4].

Plasma medicine is a new field that combines plasma physics with life science and medicine [7]. Non-thermal atmospheric-pressure plasma (also called cold plasma) is generated at atmospheric pressure [7]. Non-thermal micro-plasma is characterized by producing various chemical active species (atoms and radicals) during electron impact excitation of the working gas at mild temperature and it is the basis for biomedical applications including sterilization, disinfection and medical therapies [8–11]. Non-thermal atmospheric-pressure plasma emits several kinds of reactive oxygen species (ROS) and reactive nitrogen species (RNS), such as ozone (O_3_), atomic oxygen (O), and nitric oxide (NO) [7]. The effect of ROS on cell proliferation *in vitro* has been investigated [12]. The effect of ROS was observed when endothelial cells were exposed to plasma; in this case, cell proliferation is primarily related to the release of fibroblast growth factor [13,14]. Although NO plays an important role in skin physiology, a direct employment of NO gas for medical applications is still an unsolved problem, such as the expense and potential toxicity at high NO concentration [15,16]. NO can be easily generated by non-thermal micro-plasma directly and in desired quantity at the site of use [7]. However, few studies have investigated the biological effects of micro-plasma on skin wounds *in vivo* [7,17–19]. In addition, a number of potential side effects, although minor, have been reported in response to the inclusion of ROS directly on living cells [20]. Therefore, it is important to control plasma composition and working temperature in the course of treatment.

We previously developed and employed a capillary-tube-based micro-plasma system for wound sterilization [11]. A system with controlled micro-plasma composition and working temperature is a suitable alternative for wound healing. The developed system was found to promote the proliferation and growth factor secretion of fibroblasts *in vitro* [14]. However, the biological effects of micro-plasma on wound skin tissue remain unclear. In the present study, we hypothesize that micro-plasma enhances wound healing in a skin wound mouse model. Micro-plasma with optimal temperature and composition was applied to mice harboring laser-induced wounds and wound healing was assessed.

## Materials and Methods

### Micro-plasma system and plasma diagnosis

**Fig 1:**
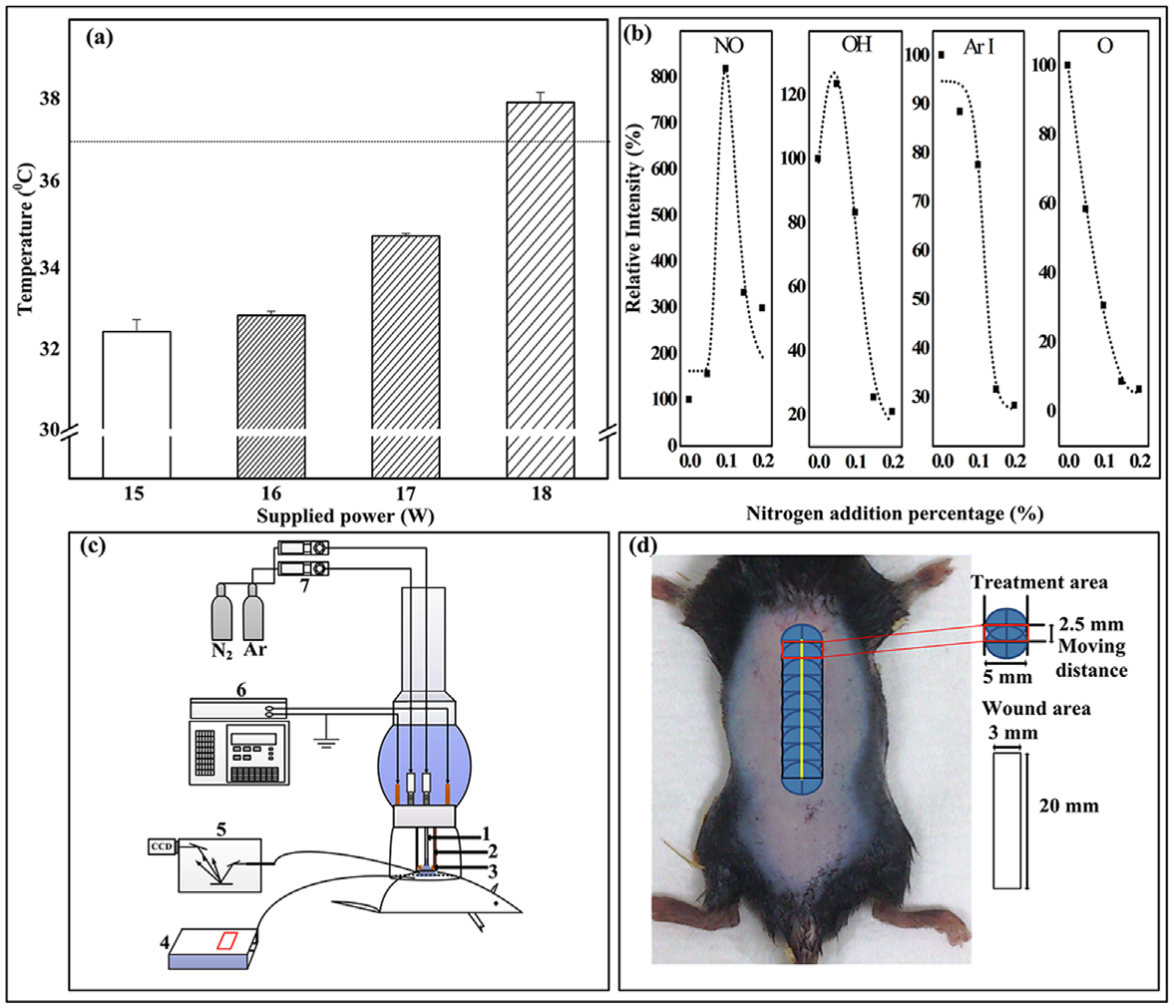
Micro-plasma diagnosis and illustrations of animal setting and treatment area. (a) Plasma plume temperature versus supply power for 0.1% N_2_/Ar micro-plasma. Error bars indicated the standard deviation of the mean for n = 6 independent experiments. (b) Relative intensities of plasma species versus percentage (0, 0.1 and 0.2%) of N_2_ addition in Ar plasma, as determined from optical emission spectrum (OES). (c) Illustrations of micro-plasma system, target mouse, and OES system (1. hollow stainless steel inner electrode, 2. dielectric quartz tube, 3. outer copper electrode, 4. fiber optic thermometer, 5. OES device, 6. radio frequency power supply, and 7. mass flow controller). (d) Dorsal region treated with laser fluences can generate wound with ablative 3 mm × 20 mm column, penetrating the mid dermis. 5 mm × 2.5 mm treatment area was exposed to 0.1% N_2_/Ar micro-plasma.

In our previous report, we developed a capillary-tube-based micro-plasma system with controlled micro-plasma composition and working temperature [10]. The micro-plasma composition and working temperature as shown in Fig 1(a), using the optical emission spectra of 0, 0.1, and 0.2% mixed nitrogen (N_2_) and Argon (Ar) micro-plasma as examples; NO-γ (237 nm, denoted as NO; according to International Commission on Non-Ionizing Radiation Protection (ICNIRP) guidelines, Ultraviolet radiation (UVR) exposure of unprotected skin should generally not exceed of 30 J/m^2^ in the spectral region of 180 to 400 nm; OH (306 nm); Ar I (750 nm); and O (777 nm) were identified. The intensity of each plasma species with respect to Ar I was used as the reference (100%). The NO level with very low UVR intensity [11] increased significantly with the addition of 0.1% N_2_, whereas the OH emission intensities (from atmospheric moisture) slightly decreased. However, the Ar I and O (from air) levels decreased with increasing addition of N_2_.

The plasma plume temperature was estimated using a fiber optic thermometer (Luxtron 812, Santa Clara, USA). The fiber was placed on an XY coordinate table. The distance from the fiber to the micro-plasma jet nozzle was ≈ 4 mm. The temperature of micro-plasma was measured at an applied power of about 15–18 W (Fig 1(a)) and an Ar flow rate of 5 slm. To generate the micro-plasma, the following settings were used: a supply power of 17 W, 0.1% N_2_ in Ar (Fig 1(b)), and an average temperature of below 40°C (line marked 37°C in Fig 1(a)).

### *Ex Vivo* Experiment for NO oxidation products in the plasma-treated skin tissue lysate

The difficulties to quantify the NO absolute concentration *in vivo* have been reported [21]. Nitrite is the product after NO oxidation; a high correlation between NO concentration and nitrite concentration in biological samples has been established [22]. Presumably, nitrite can be measured using the Griess assay (Promega, Madison, MI, USA) [16,23] to quantify NO accumulation *in vivo*.

**Fig 2:**
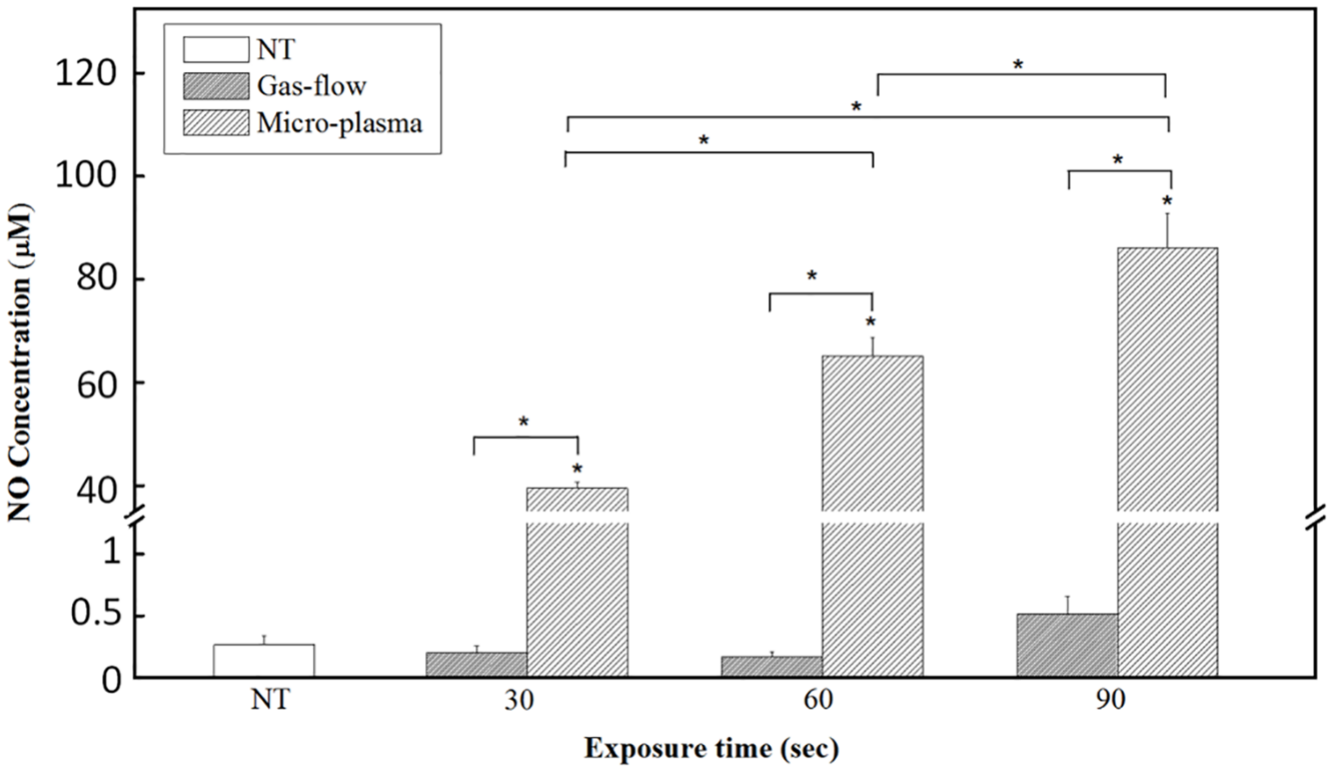
NO production in micro-plasma treated tissue lysate. NO concentrations under micro-plasma and gas flow exposure for 30, 60, or 90 secs, respectively. The NT group was as an experimental control, because pure gas flow with varied exposure time did not have significant change with NT. NO concentrations are expressed as the means ± standard deviation of the mean (SD) (*p < 0.05 compared with all other groups).

To measure NO accumulation in the plasma-treated skin tissue, at first, the dorsal skins (3 mm × 20 mm) were isolated from the back of mice after sacrificed. The skin samples were frozen in liquid nitrogen and then homogenized by a homogenizer (935C Cobb Place Blvd. Kennesaw, Geogia, USA) in 1x lysis buffer (Biochain Inst, Inc. Eureka Drive, Newark, CA, USA). The extracted tissue was cleared by centrifugation a 16000g for 20 min at 4°C (Centrifuge 5415R, Hamburg, Germany). Then 50 μl aliquots of tissue lysate were added to each well of 96-well plates and then exposed to micro-plasma or gas flow for 30, 60, or 90 sec. 50 μl of sulfanilaminde solution (Promega) was dispensed into the samples and incubated for 10 min at room temperature (while protected from light). 50 μl of N-1-nathylethylenediamine dihydrochloride solution (Promega) was then dispensed into the samples and continuously incubated for 10 min at room temperature (while protected from light). The absorbance at 500 nm for these samples was measured using microplate reader. The nitrite content (μmol NO_2_/L) was then quantified by spectrometry using sodium nitrite as the standard. Then the absolute concentration of NO (μmol NO/L) was calculated (Fig 2) [21].

### Animal model and study groups

All experiments were approved by the Institutional Animal Care and Use Committee (IACUC, Approval No. 101281) at the Laboratory Animal Center of National Cheng Kung University, Tainan, Taiwan. Eight-week-old C57BL/6JNarl male mice were obtained from the National Laboratory Animal Center, Taipei, Taiwan. The mice had a mean body weight of 24.3 ± 2 g at the beginning of experiments.

Initially, the mice were pre-anesthetized with 2% isoflurane inhalation (USP, Baxter, Guayama, USA), and all efforts were made to minimize suffering. The mice were stretched and fixed using adhesive tape to level their dorsal skin. A wound was created in the dorsal skin of the mice using a fractional CO_2_ laser (CICU, Ilooda Co., LTD, Korea). A CO_2_ laser is a useful tool for establishing an animal wound model [24]. The biochemical changes noted after CO_2_ laser resurfacing present a well-organized and highly reproducible healing response. Laser fluences were delivered on the central region of dorsal skin with a pulse energy of 39 mJ. The parameters of the laser device were set as follows: 3rd overlap (degree), 0.3-mm laser point distance, and 3 repeats (1 second / repeat) (S1 Fig). According to the manufacturer, the region treated with these parameters can generate a wound with an ablative column width of 3 mm, a length of 20 mm, and mid-dermal penetration, which is defined as a partial thickness skin defect [25,26]. No dressing was used to cover the dried wound area. After wound area was created, the wound area was shown as the red-lined rectangle (3 mm × 20 mm) in Fig 1(d). For the micro-plasma treatment on the wound region, the micro-plasma can be generated in a treatment area with a circle in a diameter of 5 mm shown as the blue circle in Fig 1(d). Due to the treatment area could not cover the whole wound extent, the treatment process was completed by moving from the top of wound (1 min for each blue circle) to the lower ones with a moving distance of 2.5 mm, as indicated in Fig 1(d).

The mice were randomly divided into the following groups: mice with laser-induced wound sites (i) without plasma treatment (denoted as non-treatment; NT), (ii) with a single plasma treatment (PT) on the day of wound generation (defined as day 0) (denoted as PT1), and (iii) with three plasma treatments once a day on days 0, 1, and 2 after wound generation (denoted as PT3). The micro-plasma device with respect to a target mouse is illustrated in Fig 1(c). The area of the laser-induced wound (3 mm × 20 mm), micro-plasma treatment area (eight overlapping regions; 5 mm × 2.5 mm each), and exposure time (1 min per overlapping region) are shown in Fig 1(d).

After treatment, the mice were kept in cages and put in animal rooms with a 12-h light/dark cycle and maintained at room temperature (23–24°C). The mice had free access to water and standard laboratory chow. The mice were housed in separate cages to protect them from bites and to avoid fighting after wounds were inflicted in their dorsal region. The condition of the mice was monitored at least once per 8 hr after wound induction. When the mice were shown behavior for pain or distress after wound induction, the trained individuals would treat mice with Butorphanol (intramuscular injection, 0.1 ~ 0.5 mg / 100 g body weight) to minimize suffering of the mice. There was no unintended death of mice during this study. At the indicated time point, the mice were euthanized by trained individuals using CO_2_ inhalation. The wound-region skins of mice in each group were harvested for further assessments.

### Wound closure kinetics

**Fig 3:**
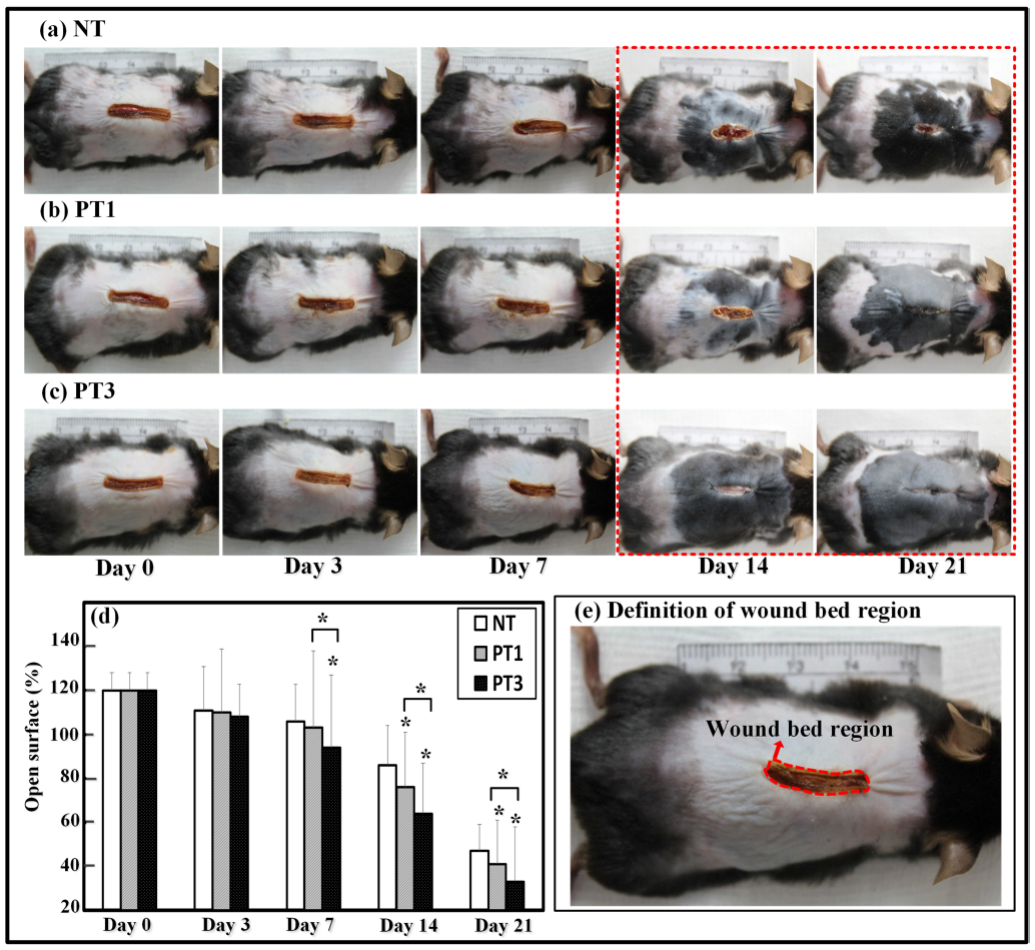
Wound closure kinetics study. Laser-induced wounds were created on day 0 and measured over 21-day observation period. Representative photographs for (a) NT, (b) PT1, and (c) PT3. (d) Measurements of open surface in mice conducted on days 0, 3, 7, 14, and 21. Percentages of open surface are expressed as the means ± standard deviation of the mean (SD) (*p < 0.05 compared with all other groups) as a function of post wounding day (n = 6).

For wound closure kinetics, the wound bed regions of six mice in each group were compared. Digital photographs of the wounds in each mouse captured on days 0, 3, 7, 14, and 21 were compared with the initial photographs taken on day 0. Based on the study Brem [27], the site of the wound bed was defined as shown in Fig 3(e). Digital planimetry software (ImageJ, National Institutes of Health, Bethesda, USA) was used to quantify the area change in the wound bed, also as shown in Fig 3(e). The investigators measuring wounds were blinded to the groups and treatment conditions.

### Non-invasive assessment

For non-invasive assessment, the wound bed regions of six mice in each group were investigated. The images obtained from optical coherence tomography (OCT, OCS1300SS, Thorlabs, USA) were along the transverse plane of the mice and had dimensions of 6 mm (X) × 6 mm (Y) × 3 mm (Z). Blood flow in the wound area was measured using a laser Doppler scanner (Moor LDIS, Moor, UK). For each measurement, a signal was generated that scales linearly with tissue perfusion, defined as the product of the blood cell velocity and concentration. This signal was presented as a two-dimensional color image on a computer screen. The produced colors illustrated the spectrum of perfusion in the wound, where dark blue depicts the lowest level of perfusion and red the highest. The system simultaneously produced a photograph, allowing for a direct anatomical comparison of the corresponding area of the laser-induced wound. For each mouse, the region of interest (ROI) was selected after the image was imported into the Moor LDI Image Review software and an area including the wound sites (3 mm × 20 mm) was scanned. The scans were performed on days 1 and 21 after wound generation to assess blood flow at the wound sites.

### Hematoxylin-eosin staining and immunohistochemical analysis

At the indicated time point, six mice in each group were sacrificed and partial thickness skin samples including the entire wound and 5 mm of the surrounding unwounded skin margin were excised using a disposable microtome blade. Then, the skin samples were bisected through the center of the lesion to obtain the largest diameter of the wound margin and immediately fixed by immersion in 4% formaldehyde prepared in 1× phosphate-buffered saline (pH 7.2), followed by routine histological processing and paraffin embedding. The samples were serially sectioned at a thickness of 3 μm and thereafter deparaffinized and rehydrated. The expression patterns of inflammatory cells and granulation tissue were discerned in the wound sections using hematoxylin and eosin (H&E) staining. For immunohistochemical (IHC) analysis, sections were pre-incubated with a mouse/rabbit polydetector peroxidase block for blocking endogenous peroxidase activity (BioSB, Inc., Santa Barbara, CA, USA) and then incubated with an monoclonal antibody against MMP-3 (dilution 1/100; ab53015, Abcam, Cambridge, MA, USA) and an polyclonal antibody against laminin (dilution 1/1000; AB2034 Sigma-Aldrich, St. Louis, MO, USA), which was detected with a mouse/rabbit polydetector horseradish peroxidase secondary antibody (BioSB, Inc., Santa Barbara, CA, USA). Nuclei were stained with hematoxylin. Digital images were acquired with a microscope digital camera (DFC 450 C, Leica, Wetzlar, Germany) attached to a light microscope (DM IRB, Leica, Wetzlar, Germany). The images of MMP-3 or laminin stained sections was quantitated using Image-Pro Plus 5.0 software (Media Cybernetics, Silver Spring, MD, USA). Briefly, the images of stained sections in each group were examined by Image-Pro Plus 5.0 software, and the intensity of DAB-stained areas (brown areas) was quantitated.

### Statistical analysis

Each experiment was repeated at least three times. The data were expressed as the means ± standard deviation of the mean (SD) of the combined data from each experimental replicate. The data were analyzed using SPSS (version 17.0, IBM, Armonk, NY, USA) to establish significant difference between data points. The *Kolmogorov-Smirnov* and *Shapiro-Wilk* methods were firstly used for testing the normal distribution of our data. Probability (*p*) values of > 0.05 were considered normally distributed. After the normal distribution of our data was confirmed, the statistical significance in each group was evaluated using one-way analysis of variance (ANOVA), and multiple comparisons were performed using *Scheffe*’s method. Probability (*p*) values of ≤ 0.05 (*) and ≤ 0.01 (**) were considered statistically significant.

## Results

### Increased NO concentration in micro-plasma treated tissue lysate

To determine the NO accumulation in skin tissue after micro-plasma treatment, nitrite which the only one stable end-product of the autoxidation of NO in the aqueous tissue lysate was measured by the Griess assay. The NO concentration in the micro-plasma treated tissue lysate sharply increased with the addition of micro-plasma exposure time. The NO concentrations were 39.48 ± 1.26, 65.13 ± 3.49, and 86.12 ± 6.68 μM for micro-plasma exposure times of 30, 60, and 90 sec, respectively. The NO concentration for the native skin (NT group) was 0.27 ± 0.07 μM. The NO concentrations in the tissue lysates of mice skin subjected to the gas flow exposure did not show significant change compared to the NT group (Fig 2).

### Micro-plasma treatment promoted wound closure in wound bed region

To investigate the effect of micro-plasma treatment on wound healing, the wound closure kinetics of the wound bed region after micro-plasma treatment was investigated. The wound bed region was measured at each indicated time point. The wound bed region is defined in Fig 3(e). In comparison to the NT group (without micro-plasma treatment), the open surface of the wound bed region in the PT3 group was reduced significantly on day 7 (Fig 3(d)). The open surface of the wound bed region in the PT1 and PT3 groups was reduced significantly from day 14 to 21 (Fig 3(d)) compared to that of the NT group. The PT3 group showed more pronounced wound bed region closure than did the PT1 group from day 7 to 21 (Fig 3(d)). These results suggest that micro-plasma treatment promoted wound closure in the wound bed region.

### Micro-plasma treatment promoted tissue regrowth in wound bed region

**Fig 4:**
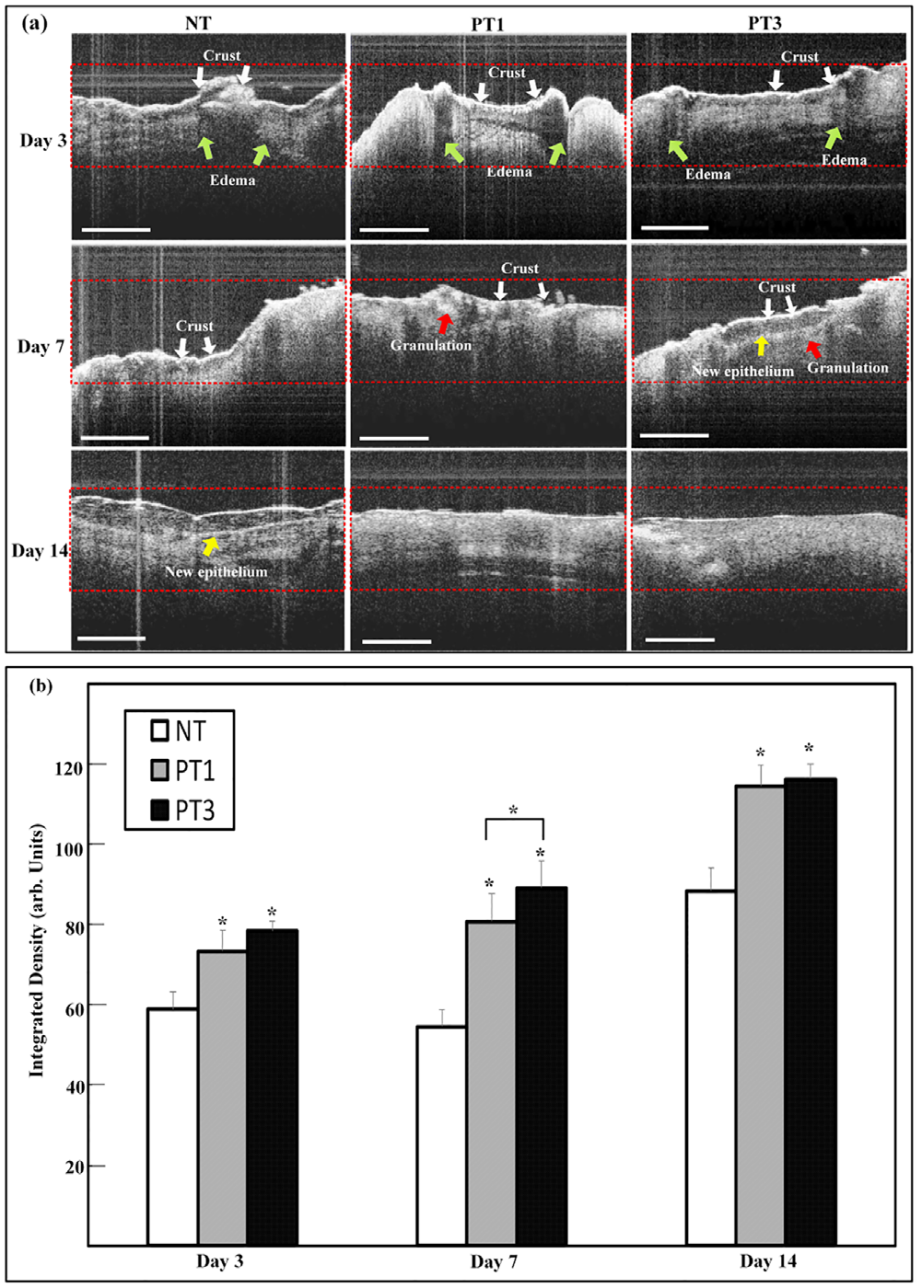
The OCT images of wound tissue after micro-plasma treatment. (a) Representative OCT images were acquired on days 3, 7, and 14 for each group. Specific characteristics of OCT images were identified as crust (white arrow), edema (green arrow), granulation (red arrow), and new epithelium (yellow arrow). (b) The backscattered light of OCT images was measured in ROI. Arbitrary units of integrated density are displayed as the means ± standard deviation of the mean (SD) (*p < 0.05 compared with all other groups) as a function of post wounding days (n = 6). Scale bar = 200 μm.

To investigate the effect of micro-plasma treatment on new tissue formation in the wound bed region, the integrated density in the wound bed region after micro-plasma treatment was evaluated from the OCT image. The architectural changes were evaluated in each group. The results showed that more homogenous structure under wound bed region after micro-plasma treatment (Fig 4). The more homogenous and parallel structure under wound bed region can be found in micro-plasma treatment groups in PT3 group on day 7&14. New tissue formation in the three groups was also quantified by measuring the integrated density in the OCT image. In comparison to the NT group, the integrated density of the wound bed region in the PT1 and PT3 groups increased significantly from day 3 to 14 (Fig 4). The integrated density in the PT3 group was more pronounced compared to those for the PT1 and NT groups on day 7. The results show that micro-plasma treatment promoted new tissue formation in the wound bed region.

**Fig 5:**
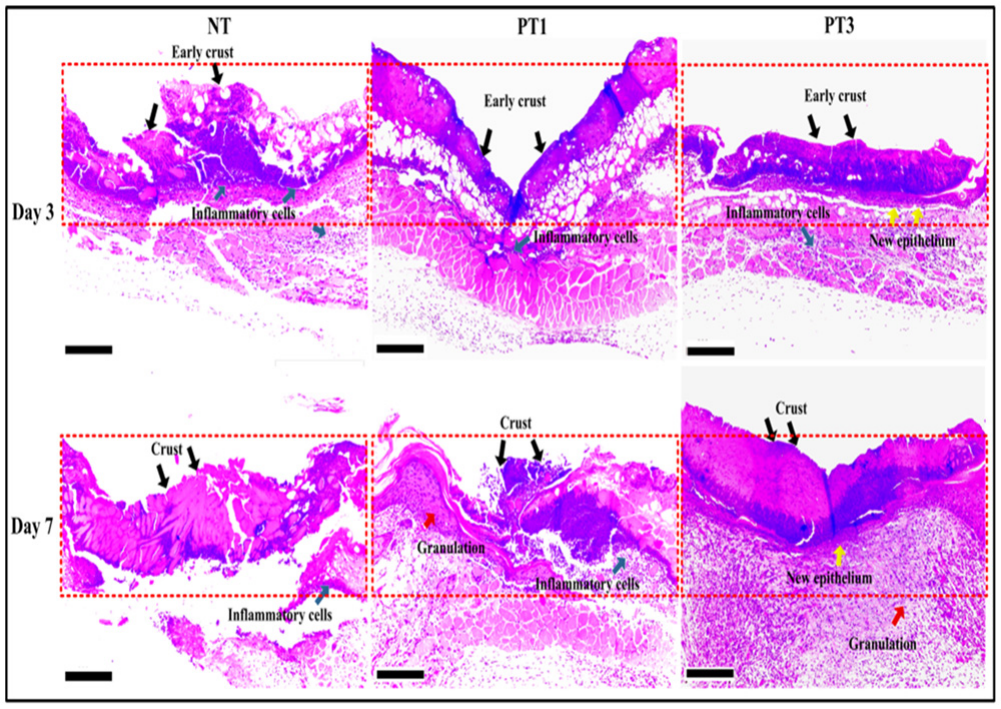
Histological observations of all groups on days 3 and 7 after wound generation. Wound tissue sections were stained with H&E. Red dotted lines demarcate central zone of laser ablation. On day 3, crusts (black arrows) could be seen in superficial layer in all groups. Inflammatory cells (green arrows) between crusted superficial layer and damaged dermal papillae were observed in all groups. Faster wound healing was found for PT3 group (indicated by earlier expression of new epithelium (yellow arrows)) compared to those for NT and PT1 groups. On day 7, crusts and inflammatory cells were still presented in the same area continuously in all groups. PT1 showed increased granulation tissue (red arrows) beneath damaged dermal papillae compared to that for NT. PT3 exhibited more visible new epithelium (yellow arrows) and granulation tissue (red arrows) in reticular dermis compared to those for NT and PT1. Scale bar = 200 μm.

Histological analysis was used for further confirmation of the OCT images shown in Fig 5. The histological analysis showed that micro-plasma treatment promoted new tissue regrowth in the wound bed region. On day 3, the H&E images showed early crust and inflammatory cells in the NT, PT1, and PT3 groups, with new epithelial cells only found in the PT3 group (Fig 5). On day 7, the PT1 and PT3 groups showed higher new tissue regrowth compared to that for the NT group. In addition to crust and inflammatory cells in the wound bed region, both new epithelium were found in the PT3 group. In the NT group, only inflammatory cells and crust were found (Fig 5). These results suggest that micro-plasma treatment promoted wound healing in the wound bed region.

### Micro-plasma treatment decreased MMP-3 but increased laminin expressions in wound bed region

**Fig 6:**
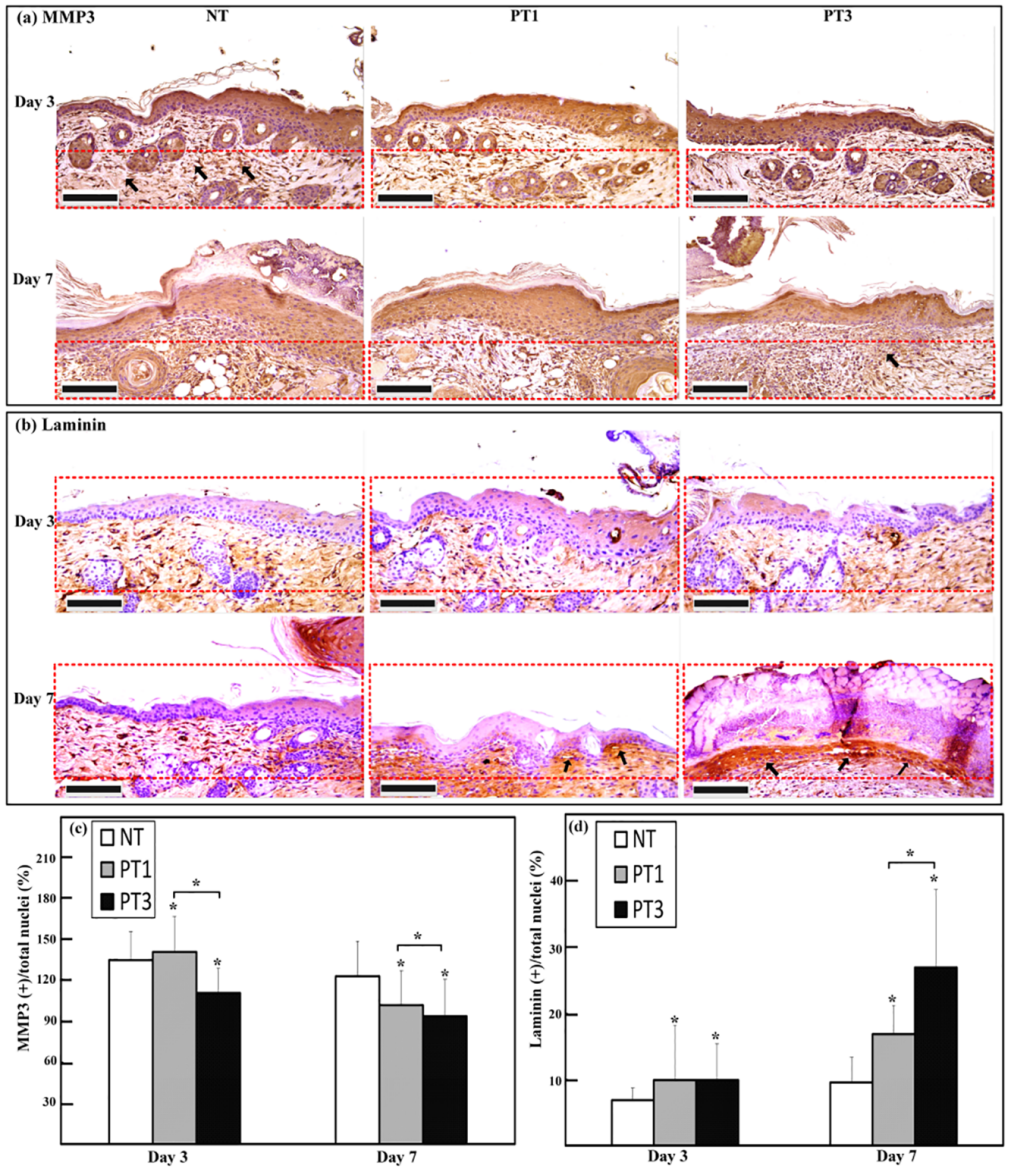
Immunohistochemical stain and semi-quantification of MMP-3 and laminin on days 3 and 7 after wound generation. (a) MMP-3 was localized in all layers of wounded skin. Red dotted lines demarcate areas of reticular dermis on side of wound margin toward central zone of laser ablation. Inside red dotted areas, weaker staining of MMP-3 appeared for PT3 compared to that for NT and PT1 on days 3 and 7. (b) Laminin was localized between basal layer of epithelium and dermal papillae, displayed with a fibrous connective tissue as dermo-epidermal junction. Red dotted lines demarcate areas between superficial layer and reticular dermis on side of wound margin toward central zone of laser ablation. Laminin immunoreactivity was more intense in PT3 than in NT and PT1 on day 7. No difference was found between PT1 and PT3 on day 3. (c) Semi-quantification analysis of MMP-3 showed significant MMP-3 protein expression localized in demarcated (a) area on days 3 and 7. (d) Semi-quantification analysis of laminin showed significant laminin protein expression localized in demarcated (b) area on days 3 and 7. MMP-3 or laminin positive stained cells of total nuclei in (c-d) are shown as the means ± standard deviation of the mean (SD) (*p < 0.05 compared with all other groups) as a function of post wounding days 3 and 7 (n = 6). Scale bar = 100 μm.

The MMP-3 and laminin expressions in the wound bed region after micro-plasma treatment were investigated. The expression of MMP-3 in the wound bed region decreased after micro-plasma treatment (Fig 6(a)). The expression of MMP-3 decreased significantly on day 3 in the PT3 group compared to that for the NT group. Both the PT1 and PT3 groups showed significant decreases in MMP-3 expression on day 7 compared to that for the NT group (Fig 6(c)). The PT3 group showed a higher decrease of MMP-3 than did the PT1 group on day 3 (Fig 6(c)). In contrast, laminin was intensely expressed after micro-plasma treatment (Fig 6(b)). Both the PT1 and PT3 groups showed more pronounced laminin expression on days 3 and 7 compared to that for the NT group (Fig 6(d)). The PT3 group showed a higher increase of laminin than did the PT1 group on day 7 (Fig 6(d)).

### Micro-plasma treatment enhanced blood flow in wound bed region

**Fig 7:**
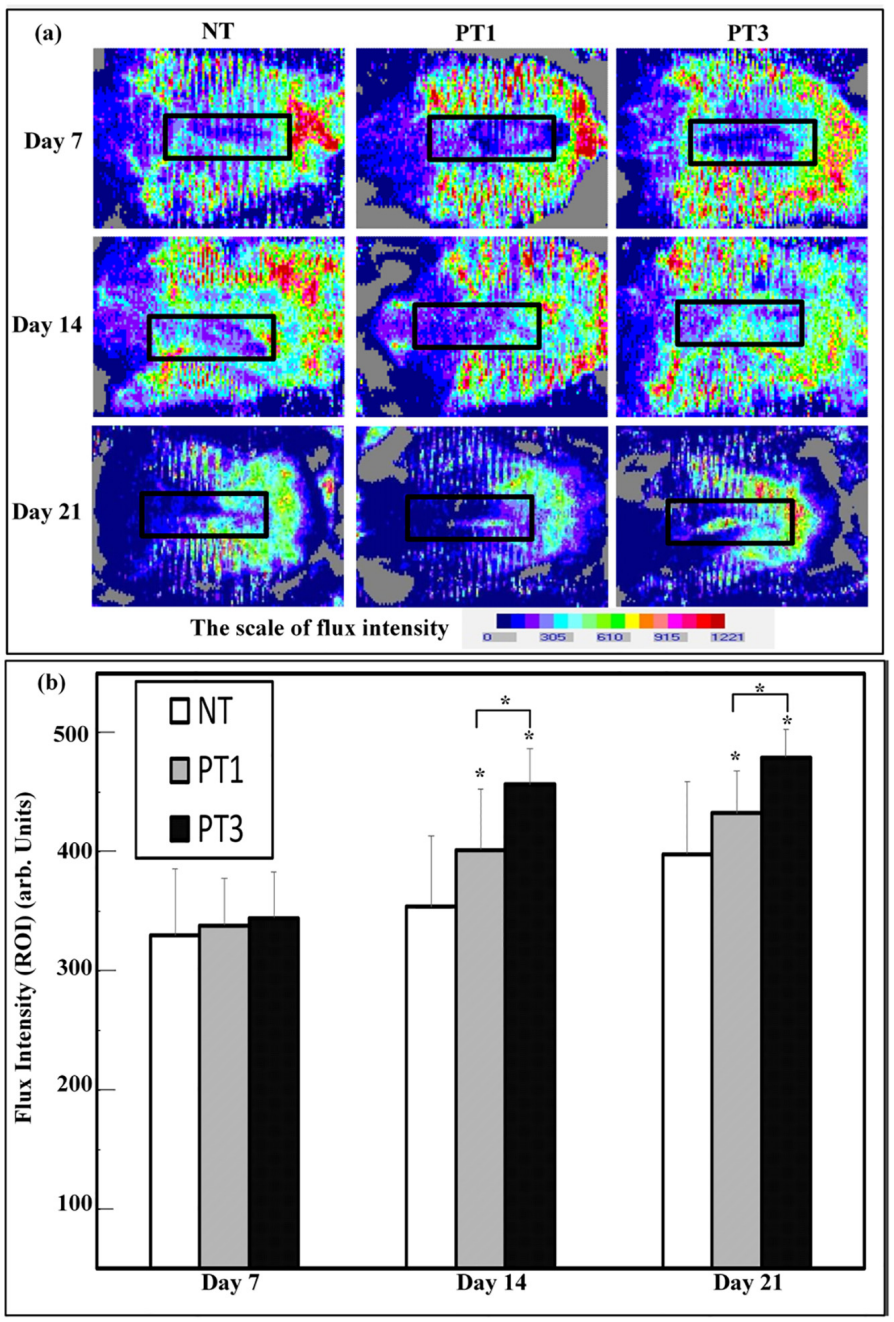
Assessment of blood flow of wound was detected by laser Doppler scanning. (a) Representative blood flow cytometry images were obtained on days 7, 14, and 21 for each group. Red areas represent increased (normal) blood flow, and blue areas represent reduced (or non-existent) blood flow. (b) Quantitative data for (a) show blood flow in ROI through flux intensity. Arbitrary units of flux intensity are expressed as the means ± standard deviation of the mean (SD) (*p < 0.05 compared with all other groups) as a function of post wounding days (n = 6).

To investigate the effect of micro-plasma treatment on increasing blood flow in the wound bed region, blood flow was detected using laser Doppler scanning (Fig 7(a)). There was an enhancement of blood flow in the PT3 and PT1 groups compared to that for the NT group on days 14 and 21. Furthermore, the PT3 group showed a higher increase of blood flow than that for the PT1 group on days 14 and 21 (Fig 7(b)). The results show that micro-plasma treatment increased blood flow in the wound bed region.

## Discussion

Wound management is still an unmet need clinically. Although micro-plasma may be used as an alternative treatment, the effect of the species generated by micro-plasma is not well defined. A system with controlled plasma compositions and working temperature is a suitable alternative for wound healing. In the present study, the efficacy of our previously developed micro-plasma system as a physical therapeutic method for enhancing the healing of ablative laser wounds in a mice model was evaluated. The wound bed region was significantly smaller in the mice treated with micro-plasma than that in the untreated mice on days 7, 14, and 21 (Fig 3). From the non-invasive OCT assessment, wounds treated three times with micro-plasma exhibited more homogenous structural change on day 7&14 than did the wounds treated only once (Fig 4). These results were also confirmed by H&E staining (Fig 5). Decreased MMP-3 and increased laminin expressions were found after three micro-plasma treatments on days 3 and 7, respectively, compared with those of the control group (Fig 6). Moreover, for wounds treated with three micro-plasma treatments, blood flow increased on days 7, 14, and 21 (Fig 7). The results suggest that micro-plasma treatment improved the healing efficacy of ablative laser wounds.

This study showed that micro-plasma treatment promotes skin wound healing *in vivo*. Micro-plasma is characterized by its temperature, types of reactive species. To maintain the non-thermal temperature as well as to adjust the plasma compositions by maintaining a relatively high NO level, it is possible to increase the overall NO concentration in the wound bed [14,20,28]. In this study, the addition of 0.1% N_2_ to Ar plasma was used to increase the NO level and simultaneously decreases the micro-plasma plume temperature (Fig 1(a) and 1(b)). After micro-plasma treatment, the target tissue does not cause any heat-associated effects. As the plasma exposure time increased, the treated site still remains within the biologically tolerable temperature range. The NO is an important molecule which has been shown to regulate many human skin process [15]. However, NO itself may also stimulate NO synthesis through inducible nitric oxide synthases in living tissue [29]. Therefore, there may be a dynamic change in NO concentration in the live skin tissue, and affects the final NO concentration. In order to reflect that the NO accumulation is generated from our micro-plasma device, we test the NO accumulation by treating the lysed skin rather than totally disintegrated skin. Our results also showed that micro-plasma treated tissue lysate have sharply increased the NO accumulation (Fig 2). These suggest that micro-plasma treatment is capable of increasing NO accumulation in wound tissue without causing undesired heat associated effect, which may damage the wound tissue.

The optical coherence tomography (OCT) has been developed for noninvasive cross-sectional imaging in biological systems [30]. The OCT is a technique that has been tested for observing the skin tissue, and has been proved capable of differentiating cutaneous structures in skin safely [31,32]. The resolution enables the visualization of architectural changes of skin tissue, but not of single cells [33]. Based on this, we test the wound bed region with OCT image after micro-plasma treatment. The results showed that new tissue regrowth in wound bed region can be found in each group by OCT image, and more gradually parallel and homogenous architectural changes of wound bed region can be found in micro-plasma treatment group (Fig 4). The result of OCT images were also confirmed H&E staining (Fig 5). Our results suggests that micro-plasma treatment promotes tissue regrowth in wound bed region.

Re-epithelialization plays an important roles in wound closure [34–36]. The enhanced NO concentration plays an important role in promoting wound healing [37]. Delayed re-epithelialization of wounds when NO synthesis is inhibited [38]. It has been reported that reactive species, including NO, result in positive effects on keratinocytes and the stimulation of angiogenesis [39]. The regeneration of a functional epidermis depends on the reconstitution of the dermal–epidermal junction (DEJ) [40,41]. Laminin is involved in re-epithelialization during wound healing, and contributes to the mechanical stability of the DEJ [42,43]. The MMP-3 enzyme degrades laminin, and MMP-3 is involved in wound healing [44,45]. In this study, we found that decreased MMP-3 expression (Fig 6a), but increased laminin expression in wound bed region after micro-plasma treatment (Fig 6b). We also found that increased blood flow in wound bed region after micro-plasma treatment (Fig 7). Our results suggest that micro-plasma treatment may promote wound healing by increasing re-epithelization and angiogenesis in wound bed region. Overall, these data support an alternative therapeutic use of a micro-plasma device with a low operating temperature and an adjustable plasma composition for wound management.

## Conclusion

Non-thermal N_2_/Ar micro-plasma exposure does not cause undesired heat associated effect, which may damage the wound tissue. Non-thermal N_2_/Ar micro-plasma exposure also showed enhanced wound healing in the wound bed region *in vivo*. These data support an alternative therapeutic use of a micro-plasma device with a low operating temperature and an adjustable plasma composition for wound management.

## Supporting Information

S1 Fig The depth analysis of laser irradiation after wounding 48 hr. Intact or wound tissue sections were stained with H&E. (a) Without laser irradiation. (b) 15.6 mJ, 1 TH, 1.0 mm. (c) 39.0 mJ, 3 TH, 0.3 mm. (d) 65.0 mJ, 3 TH, 0.1 mm. Scale bar: 100 μm.
(JPG)

S1 File The minimal data set. (RAR)
