# Supplementary figures and images for: Enhancement of Wound Healing by Non-Thermal N2/Ar Micro-Plasma Exposure in Mice with Fractional-CO2-Laser-Induced Wounds
Source: PLoS One. 2016 Jun 1;11(6):e0156699. doi: 10.1371/journal.pone.0156699 (PMC4889145; doi:10.1371/journal.pone.0156699)

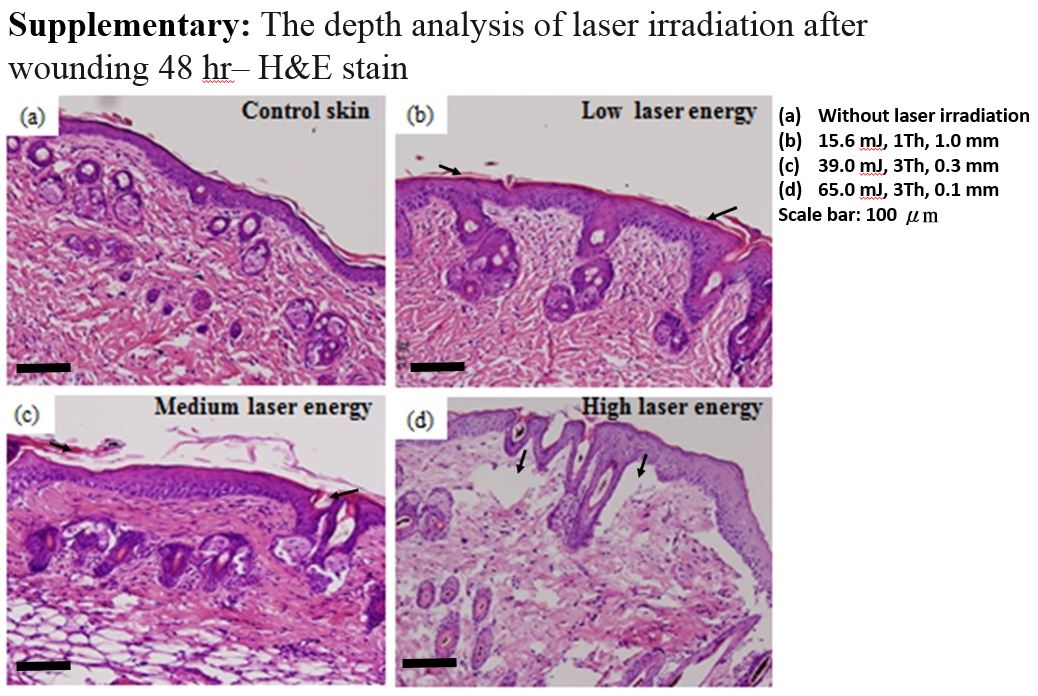

Supplement: S1 Fig — (a) Without laser irradiation. (b) 15.6 mJ, 1 TH, 1.0 mm. (c) 39.0 mJ, 3 TH, 0.3 mm. (d) 65.0 mJ, 3 TH, 0.1 mm. Scale bar: 100 μm. (JPG) [file pone.0156699.s001.jpg]
